# Mangosteen Concentrate Drink Supplementation Promotes Antioxidant Status and Lactate Clearance in Rats after Exercise

**DOI:** 10.3390/nu12051447

**Published:** 2020-05-17

**Authors:** Ching-Chien Chang, Chia-Wen Chen, Eddy Owaga, Wan-Ting Lee, Ting-Ni Liu, Rong-Hong Hsieh

**Affiliations:** 1 School of Nutrition and Health Sciences, College of Nutrition, Taipei Medical University, Taipei 11031, Taiwan; ccchang@tpcu.edu.tw (C.-C.C.); d301091008@tmu.edu.tw (C.-W.C.); mariessly@gmail.com (W.-T.L.); hungchao8016@gmail.com (T.-N.L.); 2 Department of Leisure and Recreation Management, College of Human Ecology, Taipei City University of Science and Technology, Taipei 11202, Taiwan; 3 Institute of Food Bioresources Technology, Dedan Kimathi University of Technology, Nyeri 10100, Kenya; eddy.owaga@dkut.ac.ke

**Keywords:** antioxidant capacity, exercise, lactate clearance, mangosteen, oxidative stress

## Abstract

High-strength or long-duration exercise can lead to significant fatigue, oxidative stress, and muscle damage. The purpose of this study was to examine the effect of mangosteen concentrate drink (MCD) supplementation on antioxidant capacity and lactate clearance in rats after running exercise. Forty rats were divided into five groups: N, non-treatment; C, control; or supplemented with MCD, including M1, M5, and M10 (0.9, 4.5, and 9 mL/day) for 6 weeks. The rats were subjected to 30 min running and exhaustive-running tests using a treadmill. The blood lactate; triglyceride; cholesterol and glucose levels; hepatic and muscular malonaldehyde (MDA) levels; and antioxidant enzymes, including superoxide dismutase (SOD), glutathione peroxidase (GPx), and catalase (CAT), were analyzed. The results of this study demonstrated that MCD supplementation can increase GPx and CAT activities, alleviate oxidative stress in muscle, and increase lactate clearance, and is thereby beneficial to reduced muscle fatigue after exercise.

## 1. Introduction

Exercise at moderate or high intensity results in the production of lactate in skeletal muscle and the subsequent elevation of its concentration in blood plasma. Lactate is derived from the glycolytic reduction of pyruvate, a reversible reaction that is catalyzed by lactate dehydrogenase (LDH). The relative amount of metabolism via LDH in the forward (pyruvate + NADH + H^+^ → lactate + NAD^+^) and reverse directions (lactate + NAD^+^ → pyruvate + NADH + H^+^) determines to some extent whether pyruvate or lactate will be oxidized within cells. Lactate accumulation is associated with the hypoxia and oxygen debt effect following exercise. Thus, lactate plays a major role in muscle fatigue and acidosis-induced tissue damage [1,2]. However, lactate is also an important intermediary product in many metabolic processes, acting as a mobile fuel for aerobic metabolism. Lactate generation is influenced to a great extent by flux through glycolysis, the redox state, and metabolism via the LDH pathway [1,3,4]. The liver exhibits a higher net lactate clearance than any other organ, estimated at 70% of the whole body clearance [5]. The onset of muscle fatigue after exercise has also been associated with oxidative stress. Previous studies have reported significantly increased malonaldehyde (MDA) levels in muscle and the liver after exercise. Exhaustive exercise can generate reactive oxygen species (ROS), leading to significant muscle damage and inflammatory stress [6,7,8]. The major antioxidant system in vivo is comprised of superoxide dismutase (SOD) that rapidly dismutates the superoxide anion O_2_^−^ to H_2_O_2_ and is then eliminated by glutathione peroxidase (GPx) and catalase (CAT) into water [9].

Mangosteen (*Garcinia Mangostana*) is an evergreen tree, originating from the Sun hin Islands and Moluccas region of Malaysia. The pericarp of the mangosteen fruit and the yellow gum in aril are rich in active secondary metabolites, namely xanthones. The major compounds of the mangosteen xanthones comprise α-mangostin and γ-mangostin, which have shown anti-inflammatory [10,11] and antioxidant [12,13] properties in various studies. Given the importance of oxidative stress, inflammation, and muscle damage associated with high-intensity exercise, we would like to assess the effect of oral supplementation with mangosteen xanthones capable of diminishing oxidative stress generation and muscle damage. The purpose of this study was to examine the effect of mangosteen concentrate drink (MCD) consumption on lactate metabolism and antioxidant capacity in rats after exercise by treadmill running.

## 2. Materials and Methods

### 2.1. Animals

Forty male Sprague Dawley (SD) rats aged 6 weeks and weighing 250 ± 20 g were purchased from the BioLASCO Taiwan Co., Ltd. (Taipei, Taiwan). The rats were individually housed in polycarbonate cages with stainless steel lids in an air-conditioned room (23 ± 2 °C, 65% ± 5% RH) with a 12 h light–dark cycle and free access to a standard chow diet (Labdiet^®^ 5001, Land O’Lake Inc., St. Louis, MI, USA) and water for one week before the experiment. All the animal experimental procedures were approved by the Institutional Animal Care and Use Committee of Taipei Medical University, Taipei, Taiwan.

### 2.2. Diets and Experimental Groups

Forty rats were divided into five groups, including non-treatment (N), control (C), 1× dose (M1), 5× dose (M5), and 10× dose (M10) groups; there were eight rats in each group. The N group had a non-exercise test and MCD supplementation; the C group only had exercise tests; the other groups all had exercise tests and MCD supplementation. All the groups were fed with a 28 g standard chow diet every day and additional appropriate dosages by p.o. between 1 and 5 p.m. (M1, 0.9 mL/day; M5, 4.5 mL/day; M10, 9 mL/day) for 6 weeks. The mangosteen concentrate drink was purchased from the LordDuke Biotechnology Co., Ltd. (Taipei, Taiwan). In brief, the MCD was processed by blending whole mangosteen fruit including pericarps, resulting in a red-brown drink. The density of the MCD was 1.1 g/mL and it contained (per gram) 7 kilocalories, 6.36 mg protein, 174.5 mg carbohydrate, 8.2 mg dietary fiber, and 1.44 mg alpha-mangostin. Baseline fasting blood samples were collected from the tail vein of all rats after anesthetization. Additional fasting blood samples were collected after 3 and 6 weeks of MCD supplementation. The tested rats fasted for 12 h before the blood sample collection. Furthermore, the blood samples of the rats, except the N group, were collected immediately after a 30 min treadmill running exercise and after a 30 min post-running rest at week 2 and week 4. At the end of experiment, all the rats (except the N group) were subjected to an exhaustive running test to determine the exhaustion time, and then blood samples of all the groups were drawn by exsanguination from the abdominal aorta and the liver and gastrocnemius muscle were collected. The blood lactate levels were measured immediately after the blood collection and all the collected samples were frozen at −70 °C for other analyses.

### 2.3. Treadmill Running Exercise Test

Treadmill running exercise was performed using a motor-driven treadmill comprising a 60 W electric motor, 120 × 60 cm 6-ways running surface, 0–30 m/minute speed control, and 0°–30° incline angle control. A shock grid at the rear end of the motor-driven treadmill supplied a mild electric stimulation to the feet (450 V, 10–15 mA) when the rats refused to run at the set treadmill rate. Briefly, the protocols of the habituation and the exercise tests were modified from those of Soya et al. [14]. The rats (except the N group) were initially acclimated to run for 2 days with a graded increase in velocity (from 10 to 25 m/min) within 15 min on the treadmill prior to the 30 min running and exhaustion tests. In the running tests, the treadmill speed was set from 5 to 20 m/min within the initial twenty mins and then set up at 20 m/min (5° upward tilt) for three min, 20 m/min (10° upward tilt) for three min, and finally 25 m/min (10° upward tilt) until 30 min or exhaustion.

### 2.4. Determination of Exhaustion Time

After 6 weeks of MCD treatment, the exhaustion time of the test groups was determined by counting the running time until exhaustion. The rats were considered exhausted when they still refused to run despite receiving stimulation on their feet. The time to exhaustion was defined as the time between the commencement of running and the failure of rats to keep up with the speed of the treadmill.

### 2.5. Analysis of Plasma Glucose, Cholesterol, Triglycerides, and Lactate

Appropriate commercial kits (Randox Laboratories, Crumlin, UK) were used to determine the concentrations of glucose, cholesterol, triglycerides, and lactate.

### 2.6. Analysis of Antioxidant Enzymes Activities and Malondialdehyde (MDA) Levels

The gastrocnemius and liver samples were homogenized in a 0.1 M potassium phosphate buffer (pH 7.4) with a homogenizer (Qiagen^®^ Tissuelyser II, Hilden, Germany). The supernatants were collected after being centrifuged at 10,000× *g* (10 min., 4 °C). The protein contents of tissue samples were measured using a Pierce^®^ BCA protein assay kit (Thermo Fisher Scientific Inc., Waltham, MA, USA) prior to analyzing the antioxidant enzyme activities and MDA levels. The activities of SOD, GPx, and CAT were analyzed using commercial kits (RANDOX Laboratories Ltd., UK). The MDA level was evaluated using a thiobarbituric acid reactive substances assay kit (Cayman Chemical, Ann Arbor, MI, USA). The TBARS data were expressed as nanomoles of MDA per milliliter (in plasma) or per milligram of protein (in tissues).

### 2.7. Statistical Analysis

A statistical analysis of the data was performed using the SPSS statistical software (version 19.0). The results were expressed as the mean ± SD. The differences between the parameters were analyzed by a one way analysis of variance followed by Duncan’s multiple range test for a post hoc analysis. The differences were considered significant at a *p* < 0.05.

## 3. Results

### 3.1. Body Weight and Fasting Plasma Glucose, Lipids, and MDA Levels

As shown in Supplementary Table S1, no significant changes were observed in the body weights between all the treatment groups in the study. Further, Supplementary Table S2 shows there were no significant differences in the diet consumption amounts between the various treatment groups during the experimental period. Before the MCD administration, the fasting plasma glucose and lipid levels of the rats were not significantly different between all the groups (Table 1). After 3 weeks of MCD consumption, the fasting plasma triglycerides concentrations of the rats in the C, M1, M5, and M10 groups were 29.5%, 28.7%, 40.9%, and 38.5%, respectively—significantly lower than levels of the N group (Table 1). Furthermore, the triglycerides concentrations of the M5 and M10 groups were significantly decreased by 16.1% and 12.8%, respectively, compared with the C group. The total cholesterol concentrations of the M10 group were significantly decreased by 12.8% compared with the N group and significantly decreased by 6.3% compared with the C group.

After 6 weeks of treatment, the fasting plasma glucose concentrations in the C, M1, M5, and M10 groups (13.4%, 10.9%, 8.6%, and 14.5%, respectively) were significantly lower than levels of the N group (Table 1). The total cholesterol (TC) concentrations were significantly decreased by 12.8%, 12.7%, 18.7%, and 29.5%, respectively. The triglycerides (TG) concentrations were significantly decreased by 33.6%, 34.2%, 39.6%, and 45.1%, respectively. Moreover, the TG and TC concentrations of the M10 group were significantly decreased by 17.2% and 19.1%, respectively, compared with those of the C group. These results reveal that short periodic running exercise treatment and daily MCD administration can improve glucose and lipid metabolism in vivo.

### 3.2. Plasma Lactate Levels of the Rats in the 30 Min Running Test

After 2 weeks of MCD supplementation, the plasma lactate levels of the M1, M5, and M10 groups whether before running, after running, or after rest were not significantly different when compared with the levels in the C group (Table 2). However, after 4 weeks of treatment, the plasma lactate levels of the rats in the M5 and M10 groups after a post-running rest were significantly decreased by 19.0% and 10.9%, respectively. During the post-running rest, the reduction in lactate levels in the M5 and M10 groups was significantly elevated by 12.5% and 25.5%, respectively, compared with the C group. These results reveal that MCD consumption promoted the elimination of plasma lactate during rest after exercise.

### 3.3. Running Time and Lactate Levels of the Rats in the Exhaustive Running Test

After 6 weeks of MCD supplementation, the running times of the testing groups in exhaustive test were not significantly different. Nevertheless, the running time of the M10 group was increased by 9.5% compared with the values of the C group (Table 3). The plasma lactate levels of the M1, M5, and M10 groups before/after running and after resting were not significantly different when compared with the C group. However, the lactate reduction in the M10 group during resting was significantly increased by 27.0% compared with the C group (Table 3).

### 3.4. Hepatic and Muscular MDA Levels and Antioxidant Enzymes Activities of the Rats after the Exhaustive Running Test

The hepatic MDA levels of the rats in the M5 and M10 groups were significantly decreased by 17.3% and 15.4%, respectively, compared with the C group after the exhaustive test (Table 4). Moreover, the hepatic SOD activities of the rats in the M5 and M10 groups were significantly decreased by 16.8% and 19.5%, respectively, compared with the C group; as well, the hepatic CAT activities decreased by 17.7% and 14.8%. The muscular MDA levels of the rats in the C, M1, M5, and M10 groups were not significantly different. However, only the muscular MDA level of the C group was increased by 19.6% compared with N group, although this was not significant (Table 4). Furthermore, the muscular SOD activity of the rats in the M10 group was significantly decreased by 17.3% compared with the C group, but the hepatic GPx activities of the M5 and M10 groups were significantly increased by 16.8% and 19.5%, respectively. The results of the N and C groups reveal that exhaustive running could increase the hepatic and muscular MDA levels, although not significantly, and significantly increase the antioxidant enzymatic activities. Moreover, the consumption of MCD could eliminate the hepatic and muscular MDA levels.

## 4. Discussion

### 4.1. Effect of MCD Supplementation on Fasting Plasma Biochemical Parameters

At the end of this study, the fasting plasma TG and TC of the M10 group were significantly decreased compared with the C and other MCD supplement groups. The results showed that MCD supplement could improve glucose and lipid metabolism in vivo. This is in agreement with findings of a previous human study that showed 8 weeks administration of capsules containing mangosteen fruit powder significantly decreased the total cholesterol and triglyceride levels [15]. In a previous in vitro study, α-MG from the hulls of mangosteen was found to inhibit fatty acid synthase (FAS) [16]. Moreover, a previous study suggested that α-MG exerts anti-obesity effects, ameliorates insulin resistance, and reduces TG and total cholesterol levels in high fat diet-induced obese mice by activating the hepatic AMPK, SirT1, and PPARγ expression, which has been found to increase the rates of fatty acid oxidation and repress lipogenesis [17]. In order to further elucidate the underlying effect of MCD on lactate clearance, we suggest that further studies include the hepatic concentration of TG and TC.

### 4.2. Effect of MCD Supplementation on the Lactate Clearance of the Rats after Running Tests

In this study, plasma lactate clearance was significantly increased in the MCD supplementation groups after running tests. The M10 group had longest running time and highest rate of lactate clearance compared with the other groups after the exhaustive exercise test. Previous studies showed that the production and removal of lactate in vivo occur via a reversible redox reaction catalyzed by LDH, which is located in the cytoplasm [18,19]. The main source of lactate production in humans includes intracellular glucose (65%) and alanine (16%–20%), because glycolysis involves a series of steps by which glucose is metabolized into pyruvate in the cytoplasm and later converted to lactate. Under hypoxic conditions, pyruvate is reduced to lactate, whereas the reduced nicotinamide adenine dinucleotide (NADH) is oxidized to NAD^+^ [20]. The lactate threshold has been defined as the work rate or oxygen uptake beyond which the blood lactate concentration begins to rise more rapidly [21]. In resting muscle and the post-absorptive state, blood lactate from skeletal muscle provides about 40% of lactic acid in the systemic circulation and the net lactate extraction by the liver is partly aimed at the production of endogenous glucose; however, some researches showed that in active muscles, either lactate release or extraction are comparable to the levels in muscles at rest [22,23]. The conversion of lactate into pyruvate via LDH catalytic oxidation is an important step towards eliminating accumulated lactate in the cytoplasm [24]. In addition, the production of endogenous glucose through gluconeogenesis can also contribute to lactate clearance. Some studies have indicated that hepatic lactate uptake and gluconeogenesis are increased during the exercise period [24,25,26]. Endurance training can reduce lactate production in exercise muscle and release to blood, because exercise may enhance the oxidation efficiency of the respiratory chain, hence reducing non-oxidative glycolysis and lactate formation [27,28,29].

### 4.3. Effect of MCD Supplementation on Hepatic and Muscular Antioxidant Enzymes Activities and the MDA Levels of the Rats after Running Tests

In the present study, the muscular MDA levels of the MCD consumption groups were decreased compared with the C group, although these were not significant after the exhaustive test. However, the hepatic MDA levels of the M5 and M10 groups declined markedly compared with the C group. In this study, it was found that exhaustive exercise caused increased muscular MDA levels but did not affect the hepatic MDA levels. Vasilaki et al. and Zheng et al. have represented that endurance and high-strength or exhaustive exercises cause muscle damage accompanied by oxidative stress and inflammation, leading to muscle fatigue and muscle soreness [30,31]. Previous studies also have reported increased MDA contents in the muscles and liver after prolonged or intense exercise. The variation between the hepatic MDA content of the rats after exercise between the present and previous studies may have arisen from elevated plasma glucose, triglycerides, and cholesterol levels of the rats in the N group in this study. It has been reported that hyperlipidemic or hyperglycemic rats have significantly higher hepatic MDA levels [32,33].

Hellsten et al. and Viña et al. found xanthine oxidase (XO)-derived ROS as a significant source of ROS after prolonged or intense exercise as well as mitochondria-derived ROS [34,35]. The xanthine oxidase (XO) mediated the oxidation of hypoxanthine to xanthine; the further oxidation of xanthine to uric acid produces hydrogen peroxide consecutively eliminated by GPx and CAT through conversion into water. Previous studies reported that the inhibition of XO with allopurinol (a XO inhibitor) afforded protection against free radical production and tissue damage associated with exhaustive exercise in animals and humans [35,36]. The results in this study showed that the increased production of ROS during exhaustive exercise had to be eliminated by antioxidant enzymes in vivo; thus, the GPx and CAT activities of the exhaustive exercise groups were more increased than in the N group. Moreover, the results also revealed that MCD consumption could promote GPx and CAT activities, especially in muscle. This study demonstrated that MCD could improve the antioxidant capacity and lactate clearance in rats after exercise. However, the respiratory efficiency of the rats was not analyzed because of limitations of the measurement equipment. In addition, we propose further studies to determine the hepatic concentrations of xanthine compounds in relation to TG, TC, and lactate clearance in order to fully elucidate the underlying mechanism of MCD effects.

## 5. Conclusions

**Figure 1:**
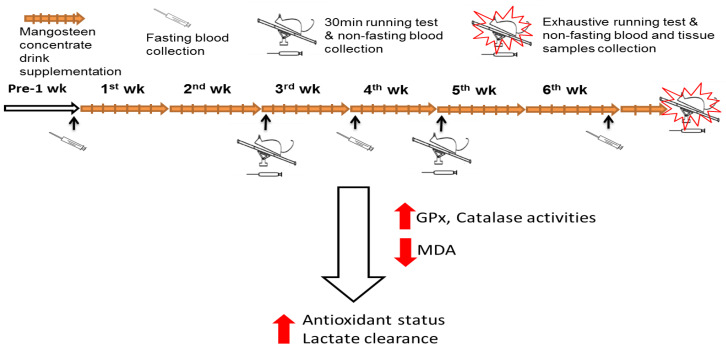
Experiment flow figure. The purpose of this study was to examine the effect of mangosteen concentrate drink (MCD) supplementation on the antioxidant capacity and lactate clearance in rats after running exercise. Forty rats were divided into 5 groups: N, non-treatment; C, control; or supplemented with MCD, including M1, M5, and M10 (0.9, 4.5, and 9 mL/day) for 6 weeks. The rats were subjected to 30 min exhaustive running tests using a treadmill and sample collections. The results of this study demonstrated that MCD supplementation can increase glutathione peroxidase (GPx) and catalase (CAT) activities, alleviate oxidative stress in muscle, and increase lactate clearance; it is thereby beneficial in reducing muscle fatigue after exercise.

This is the first demonstration of the potential utility of mangosteen concentrate drink to alleviate muscle fatigue during exercise via reducing oxidative stress and increasing lactate clearance. As demonstrated in the proposed mechanistic model in Figure 1, MCD supplementation can increase GPx and CAT activities, alleviate oxidative stress in muscle, and increase lactate clearance; it is thereby beneficial in reducing muscle fatigue after exercise.

## Figures and Tables

**Table 1:** Body weight and fasting plasma biochemical levels of the rats during the treatment period ^1,2^.

	N	C	M1	M5	M10
Initial					
Weight (g)	318.3 ± 12.3	313.4 ± 8.2	317.6 ± 14.6	312.0 ± 10.2	318.4 ± 9.1
Glucose (mg/dL)	136.6 ± 16.3	139.5 ± 14.0	141.0 ± 7.4	130.8 ± 6.8	134.5 ± 13.4
Triglycerides (mg/dL)	56.22 ± 5.39	58.40 ± 6.80	60.50 ± 5.45	57.92 ± 3.61	55.20 ± 5.26
Cholesterol (mg/dL)	65.34 ± 8.67	70.20 ± 7.15	71.40 ± 5.35	65.00 ± 3.28	65.23 ± 4.50
MDA (nmole/mL)	0.52 ± 0.03	0.52 ± 0.02	0.53 ± 0.03	0.52 ± 0.02	0.53 ± 0.02
After 3 weeks feeding					
Weight (g)	414.0 ± 14.7	417.3 ± 21.9	418.2 ± 25.8	425.1 ± 12.1	428.4 ± 20.7
Glucose (mg/dL)	154.1 ± 12.8	142.1 ± 12.1	150.8 ± 13.7	145.8 ± 12.0	139.2 ± 15.1
Triglycerides (mg/dL)	82.02 ± 7.93 ^c^	57.83 ± 9.68 ^b^	58.45 ± 3.87 ^b^	48.50 ± 8.65 ^a^	50.41 ± 9.75 ^ab^
Cholesterol (mg/dL)	64.71 ± 5.37 ^b^	60.21 ± 3.24 ^ab^	63.46 ± 4.37 ^b^	64.50 ± 3.89 ^b^	56.40 ± 6.12 ^a^
MDA (nmole/mL)	0.57 ± 0.04	0.55 ± 0.02	0.55 ± 0.03	0.54 ± 0.05	0.54 ± 0.03
After 6 weeks feeding					
Weight (g)	473.1 ± 23.5	450.3 ± 20.0	467.5 ± 29.8	470.3 ± 15.9	464.9 ± 22.6
Glucose (mg/dL)	158.0 ± 10.0 ^b^	136.8 ± 7.1 ^a^	140.8 ± 15.9 ^a^	144.4 ± 12.2 ^a^	135.1 ± 9.7 ^a^
Triglycerides (mg/dL)	87.60 ± 8.91 ^c^	58.13 ± 6.10 ^b^	57.60 ± 3.60 ^b^	52.87 ± 2.42 ^ab^	48.12 ± 3.30 ^a^
Cholesterol (mg/dL)	63.84 ± 5.91 ^c^	55.67 ± 8.38 ^b^	55.76 ± 8.30 ^b^	51.92 ± 7.48 ^ab^	45.03 ± 3.18 ^a^
MDA (nmole/mL)	0.65 ± 0.03 ^c^	0.60 ± 0.02 ^b^	0.57 ± 0.03 ^ab^	0.54 ± 0.04 ^a^	0.53 ± 0.05 ^a^

^1^ Values are means ± SD, n = 8. N, non-treatment; C, control; M1 (0.9 mL/day mangosteen concentrate drink (MCD)); M5 (4.5 mL/day MCD); and M10 (9 mL/day MCD). ^2^ Values in the same row with different letter superscripts indicate a significant change between the groups (*p* < 0.05); if without superscripts, they indicate no significant difference.

**Table 2:** Plasma lactate levels of the rats in the 30 min running period after mangosteen supplementation ^1,2^

	C	M1	M5	M10
Lactate (mg/dL)	After 2 weeks feeding			
Before running (a)	16.19 ± 2.19	15.23 ± 3.50	16.40 ± 3.31	17.13 ± 1.98
After 30 min running (b)	27.41 ± 2.16	25.88 ± 3.25	26.28 ± 2.80	27.20 ± 2.05
Lactate increase during running (b-a)	11.22 ± 3.58	10.66 ± 1.48	9.88 ± 1.44	10.07 ± 1.37
After resting (c)	18.71 ± 2.27	15.72 ± 3.39	16.53 ± 2.61	16.36 ± 1.68
Lactate decrease during resting (c-b)	9.07 ± 0.59	10.16 ± 0.82	9.92 ± 0.78	10.84 ± 1.41
Lactate (mg/dL)	After 4 weeks feeding			
Before running (a)	15.40 ± 3.56	15.08 ± 2.42	14.73 ± 2.58	14.66 ± 1.64
After 30 min running (b)	25.13 ± 2.81	24.99 ± 2.93	24.65 ± 3.11	24.53 ± 2.62
Lactate increase during running (b-a)	9.73 ± 1.13	9.91 ± 1.01	9.92 ± 1.28	9.97 ± 1.16
After resting (c)	15.54 ± 2.03 ^b^	14.67 ± 1.74 ^ab^	13.85 ± 1.15 ^a^	12.58 ± 1.94 ^a^
Lactate decrease during resting (c-b)	9.60 ± 0.90 ^a^	10.32 ± 1.59 ^ab^	10.80 ± 2.18 ^b^	12.05 ± 1.08 ^b^

^1^ Values are means ± SD, n = 8. C, control; M1 (0.9 mL/day MCD); M5 (4.5 mL/day MCD); and M10 (9 mL/day MCD). ^2^ Values in the same row with the different letter superscripts indicate a significant change between the groups (*p* < 0.05); if without superscripts, they indicate no significant difference.

**Table 3:** Running time and plasma lactate levels of the rats in the exhaustive running period after 6 weeks mangosteen treatment ^1,2^

	C	M1	M5	M10
Running time (seconds)	2331 ± 329	2361 ± 190	2354 ± 159	2585 ± 130
Lactate (mg/dL)				
Before running (a)	14.36 ± 2.58	14.00 ± 2.49	15.92 ± 3.62	16.32 ± 1.57
After 30 min running (b)	138.51 ± 41.14	152.39 ± 17.47	133.65 ± 26.65	170.61 ± 23.60
Lactate increase during running (b-a)	124.16 ± 43.20	138.39 ± 15.79	117.72 ± 28.29	154.29 ± 23.52
After resting (c)	45.69 ± 10.15	53.71 ± 17.33	37.74 ± 10.70	43.54 ± 5.97
Lactate decrease during resting (c-b)	92.82 ± 33.47 ^a^	98.69 ± 12.66 ^a^	95.91 ± 27.79 ^a^	127.07 ± 25.30 ^b^

^1^ Values are means ± SD, n = 8. C, control; M1 (0.9 mL/day MCD); M5 (4.5 mL/day MCD); and M10 (9 mL/day MCD). ^2^ Values in the same row with different letter superscripts indicate a significant change between the groups (*p* < 0.05); if without superscripts, they indicate no significant difference.

**Table 4:** Antioxidant enzyme activities and malonaldehyde (MDA) levels of the liver and muscles in rats after the exhaustive running test ^1–3^

	N	C	M1	M5	M10
Hepatic MDA (nmole/mg protein)	1.26 ± 0.09 ^b^	1.33 ± 0.11 ^b^	1.30 ± 0.15 ^b^	1.06 ± 0.05 ^a^	1.11 ± 0.09 ^a^
Hepatic SOD (unit/mg protein)	142.6 ± 19.7^b^	148.2 ± 16.9 ^b^	149.6 ± 9.9 ^b^	123.3 ± 11.0 ^a^	119.3 ± 13.6 ^a^
Hepatic GPx (unit/mg protein)	163.5 ± 7.6 ^a^	178.2 ± 9.9 ^b^	177.4 ± 10.7 ^b^	182.3 ± 19.9 ^b^	182.9 ± 11.0 ^b^
Hepatic CAT (mmole/min/mg protein)	48.5 ± 16.0 ^a^	126.8 ± 7.4 ^c^	115.5 ± 15.9 ^bc^	104.4 ± 8.8 ^b^	108.0 ± 11.2 ^b^
Muscular MDA (nmole/mg protein)	0.46 ± 0.13	0.55 ± 0.10	0.45 ± 0.06	0.45 ± 0.09	0.45 ± 0.09
Muscular SOD (unit/mg protein)	87.90 ± 7.00 ^a^	96.98 ± 11.58 ^b^	96.30 ± 5.82 ^b^	86.99 ± 6.68 ^ab^	80.20 ± 6.87 ^a^
Muscular GPx (unit/mg protein)	7.24 ± 1.49 ^a^	7.41 ± 1.08 ^a^	7.52 ± 1.20 ^a^	9.08 ± 1.72 ^b^	11.66 ± 1.85 ^c^
Muscular CAT (mmole/min/mg protein)	3.04 ± 0.55 ^a^	3.79 ± 0.34 ^b^	4.37 ± 0.97 ^b^	4.12 ± 0.57 ^b^	5.11 ± 0.64 ^c^

^1^ Values are means ± SD, n = 8. ^2^ Values in the same row with different letter superscripts indicate a significant change between the groups (*p* < 0.05); if without superscripts, they indicate no significant change. N, non-treatment; C, control; M1 (0.9 mL/day MCD); M5 (4.5 mL/day MCD); and M10 (9 mL/day MCD). ^3^ The rats in the C, M1, M5, and M10 groups were sacrificed after the exhaustive running test and 30 min post- running rest, but rats in the N group were sacrificed without running.

## Supplementary Materials

The following are available online at https://www.mdpi.com/2072-6643/12/5/1447/s1, Table S1: title. Body weight (grams) of the rats during treatment period; Table S2. Diet consumptions (grams) of the rats per week during treatment period.
