# Supplementary material for: Mangosteen Concentrate Drink Supplementation Promotes Antioxidant Status and Lactate Clearance in Rats after Exercise
Source: Nutrients. 2020 May 17;12(5):1447. doi: 10.3390/nu12051447 (PMC7284599; doi:10.3390/nu12051447)
Supplement: Supplementary file 1 [file nutrients-12-01447-s001.pdf]

Suppl. Table 1. Body weight (grams) of the rats during treatment period[1, 2]

	N	C	1X	5X	10X
**Initial**	318.3 ± 12.3	313.4 ± 8.2	317.6 ± 14.6	312.0 ± 10.2	318.4 ± 9.1
**After 1weeks feeding**	342.5 ± 14.5	344.6 ± 9.9	346.8 ± 13.9	348.8 ± 6.4	356.0 ± 13.5
**After 2weeks feeding**	380.8 ± 15.7	380.1 ± 15.3	388.6 ± 20.6	395.8 ± 10.5	395.6 ± 14.2
**After 3 weeks feeding**	414.0 ± 14.7	417.3 ± 21.9	418.2 ± 25.8	425.1 ± 12.1	428.4 ± 20.7
**After 4 weeks feeding**	429.6 ± 19.5	429.5 ± 23.2	430.7 ± 25.5	438.1 ± 12.1	441.0 ± 25.3
**After 5 weeks feeding**	449.9 ± 22.1	439.7 ± 21.0	445.8 ± 27.4	449.8 ± 13.2	449.7 ± 22.1
**After 6 weeks feeding**	473.1 ± 23.5	450.3 ± 20.0	467.5 ± 29.8	470.3 ± 15.9	464.9 ± 22.6

1. Values are means ± SD, n=8.

2. Values in the same row with the different letter superscripts indicate a significant change between groups ($p < 0.05$), if no superscript, there is no significant change. N, non-treatment; C, control; 1X (0.9 mL/day MCD); 5X (4.5 mL/day MCD) and 10X (9 mL/day MCD).

Suppl. Table 2. Diet consumptions (grams) of the rats per week during treatment period[1, 2]

	N	C	1X	5X	10X
**1st week**	178.4 ± 3.0	180.2 ± 2.9	180.3 ± 3.0	179.3 ± 2.2	179.7 ± 2.5
**2nd week**	181.8 ± 2.3	182.2 ± 2.1	181.3 ± 3.3	180.7 ± 3.2	180.3 ± 2.5
**3rd week**	184.0 ± 1.6[a]	193.8 ± 1.5[b]	193.0 ± 1.6[b]	192.9 ± 1.5[b]	193.3 ± 1.6[b]
**4th week**	187.6 ± 2.6[a]	194.0 ± 2.0[b]	192.8 ± 2.0[b]	193.4 ± 1.5[b]	192.9 ± 1.7[b]
**5th week**	189.1 ± 3.6[a]	194.1 ± 2.1[b]	194.0 ± 1.7[b]	192.4 ± 1.9[b]	191.8 ± 1.7[b]
**6th week**	190.1 ± 3.8[a]	194.0 ± 2.0[b]	193.7 ± 1.5[b]	193.5 ± 2.2[b]	192.7 ± 1.5[b]

[1.] Values are means ± SD, n=8.

[2.] Values in the same row with the different letter superscripts indicate a significant change between groups ($p < 0.05$), if no superscript, there is no significant change. N, non-treatment; C, control; 1X (0.9 mL/day MCD); 5X (4.5 mL/day MCD) and 10X (9 mL/day MCD).
